# Functional Modification of Cyanobacterial Phycobiliprotein and Phycobilisomes through Bilin Metabolism Control

**DOI:** 10.1021/acssynbio.4c00094

**Published:** 2024-07-22

**Authors:** Mizuho Sato, Takeshi Kawaguchi, Kaisei Maeda, Mai Watanabe, Masahiko Ikeuchi, Rei Narikawa, Satoru Watanabe

**Affiliations:** † Department of Bioscience, Tokyo University of Agriculture, Tokyo 156-8502, Japan; ‡ Laboratory for Chemistry and Life Science, Institute of Innovative Research, Tokyo Institute of Technology, Yokohama 226-8503, Japan; § Department of Biological Sciences, Graduate School of Science, Tokyo Metropolitan University, Tokyo 192-0397, Japan; ∥ Graduate School of Arts and Sciences, University of Tokyo, Tokyo 153-0041, Japan

**Keywords:** phycobilisome, cyanobacteria, phycoerythrin, phycocyanin, photosynthesis

## Abstract

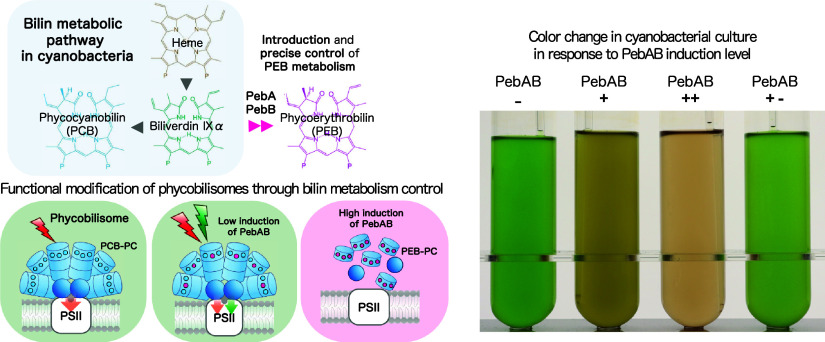

Phycobilisomes (PBSs) are light-harvesting antenna complexes in cyanobacteria that adapt to diverse light environments through the use of phycobiliproteins within the PBS structures. Freshwater cyanobacteria, such as *Synechococcus elongatus* PCC 7942, thrive under red light because of the presence of phycocyanin (PC) and its chromophore, phycocyanobilin (PCB), in the PBS. Cyanobacteria in shorter-wavelength light environments such as green light, employ phycoerythrin paired with phycoerythrobilin (PEB) along with PC in the PBS. Synthetic biology studies have shown that PEB production can be achieved by expression of the heterologous PEB synthases 15,16-dihydrobiliverdin:ferredoxin oxidoreductase (PebA) and PEB:ferredoxin oxidoreductase (PebB), leading to PEB accumulation and cellular browning. This approach is genetically unstable, and the properties of the resulting PEB-bound PBS complexes remain uncharacterized. In this study, we engineered a novel strain of *Synechococcus* 7942 PEB1 with finely tuned control of PEB biosynthesis. PEB1 exhibited a reversible change in the color of the culture from green to brown and pink based on PebA and PebB induction levels. High induction led to complete PCB-to-PEB substitution, causing the disassembly of the PBS rod complex. In contrast, low induction levels of PebA and PebB resulted in the formation of a stable chimeric PBS complex with partial PCB-to-PEB substitution. This acclimation enabled efficient light harvesting in the green spectrum and energy transfer to the photosynthetic reaction center. These findings, which improve our understanding of PBS and highlight the structural importance of the bilin composition, provide a foundation for future studies on PBS adaptation in bioengineering, synthetic biology, and renewable energy.

## Introduction

Photosynthesis is the process by which light energy is converted into chemical energy and is fundamental for sustaining life on Earth. Through evolution, photosynthetic organisms have developed optimized light-harvesting complexes to efficiently harvest light and transfer energy to photosynthetic reaction centers in their respective environments. Phycobilisomes (PBSs) are peripheral light-harvesting complexes found in the thylakoid membranes of cyanobacteria, eukaryotic red algae, and glaucophytes. PBSs enhance the light-harvesting efficiency by channeling light energy to reaction centers for subsequent conversion into chemical energy.^1,2^

Structurally, PBSs consist of rod and core subcomplexes, of which the rods are radially attached to core proteins. Several linear tetrapyrroles, known as bilins, function as chromophores and are bound to apoproteins within the rod and core subcomplexes. The rods comprise disk-like trimers, namely (αβ)_3_, that further assemble into hexamers ([αβ]_3_)_2_ which consist of several types of phycobiliproteins, including phycocyanins (PCs), phycoerythrins (PEs) and phycoerythrocyanins (PECs).^3−5^ Phycobiliproteins contain covalently bound bilins, and PCs consist of nine phycocyanobilins (PCBs) in the (αβ)_3_ disk. Rods vary depending on the organism and contain PCs only or a combination of PCs and other phycobiliproteins such as PEs and PECs. The PBS core consists of a cylindrical structure composed of allophycocyanin (APC) with six PCBs per monomer. All units (disks within the rod and rod-to-core) are connected using linker proteins.

Cyanobacteria are a monophyletic lineage of Gram-negative oxygenic photosynthetic bacteria that thrive ubiquitously and inhabit various ecological zones, including aquatic environments such as lakes, rivers, and oceans, as well as arid deserts, polar regions, and caves. In addition, they can be found in symbiosis with other organisms such as fungi, to form lichens.^6^ Physiologically, cyanobacteria produce oxygen through photosynthesis; therefore, they can create biomass using solar energy. Cyanobacteria have recently garnered attention for their potential as green cell factories for the CO_2_-neutral biosynthesis of various products.^7,8^ To date, CO_2_-derived useful material production systems, such as terpenoids and benzenoids, have been established using the metabolic engineering of the freshwater cyanobacterium *Synechococcus elongatus* PCC 7942 (hereafter *Synechococcus* 7942).^9,10^

In their natural environment, cyanobacteria employ a distinct composition of disks within their PBS rods to optimize light harvesting based on available light wavelengths. For example, freshwater cyanobacteria, such as *Synechococcus* 7942, utilize only PCs bound to PCB to efficiently absorb orange light.^11^ In contrast, the PBSs of cyanobacteria that thrive in deep-water environments have been optimized for light absorption in shorter-wavelength light conditions, such as green light (GL) environments, which are found in deep-water environments. PBS rods of cyanobacteria such as *Synechococcus* sp. WH 7803 (*Synechococcus* 7803) contains PEs that are bound to phycoerythrobilin (PEB) as well as PCs, allowing efficient light harvesting even in niche environments that are depleted of orange-to-red light.^12^ In several cyanobacteria, including *Synechococcus* 7803, two types of PEs, PEI, and PEII, are found in their PBS, containing different apoproteins for those two types.^12^

**Figure 1:**
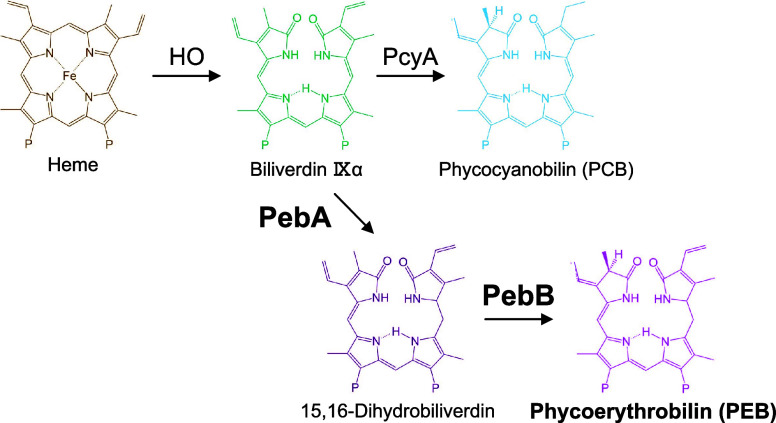
PCB and PEB metabolic pathways. Schematic overview of phycobilin biosynthesis from heme. In the cyanobacterium *Synechococcus* 7942, biliverdin IXα is converted to phycocyanobilin by the ferredoxin-dependent bilin reductase PcyA (top reaction). PCB, phycocyanobilin; PcyA, phycocyanobilin:ferredoxin oxidoreductase; PEB, phycoerythrobilin; PebA, 15,16-dihydrobiliverdin:ferredoxin oxidoreductase; PebB, phycoerythrobilin:ferredoxin oxidoreductase; and HO, heme oxygenase.

PCB and PEB are isomers derived from a common biosynthetic precursor, namely biliverdin IXα, which is synthesized from heme, and differ only in the number of conjugated double bonds forming the chromophore (Figure 1). PCB:ferredoxin oxidoreductase (PcyA) synthesizes PCBs through a region-specific reduction in biliverdin IXα.^13^ In *Synechococcus* 7803, PEB is synthesized from biliverdin IXα via the intermediary 15,16-dihydrobiliverdin (DHBV) by the sequential action of two reductases, DHBV:ferredoxin oxidoreductase (PebA) and PEB:ferredoxin oxidoreductase (PebB) (Figure 1).^12,13^ To form rod phycobiliproteins, such as PCs and PEs, the synthesized PCBs and PEBs are covalently bound to specific apoproteins (phycocyanin alpha chain [CpcA] and phycocyanin beta chain [CpcB] for PC, and phycoerythrin alpha chain [CpeA/MpeA] and phycoerythrin beta chain [CpeB/MpeB] for PE) using specialized lyases, which catalyze the binding of chromophore to apoproteins.^14−16^

Most PBS-related genes form functional clusters in the cyanobacterial genome and are proposed to be acquired and evolved through horizontal gene transfer.^12^ Nevertheless, our understanding of the potential of PBSs to accept heterogeneous components and their evolutionary capacities remains limited. An important previous study showed that the strong and constitutive coexpression of PebA and PebB (PebAB) in *Synechococcus* sp. PCC 7002 (*Synechococcus* 7002) resulted in cellular browning due to PEB accumulation.^17^ PEB binds to endogenous CpcA in *Synechococcus* 7002 without PEB lyase; however, it is unclear whether the accumulated PEB is incorporated into the PBS complex and whether the resultant PBS has light-harvesting capacity.

In the present study, we aimed to address these remaining questions and to elucidate the performance of the artificially constructed PEB-bound PBS complexes. We designed a novel recombinant strain, *Synechococcus* 7942 PEB1, which can precisely regulate PEB metabolism by controlling the expression level of *pebAB*. Using this strain, we examined the status of cells and PBS at each PEB amount. The substitution of PCB with PEB and disassembly of the PBS complex were observed under high accumulation of PEB. On the other hand, under low PEB conditions, the partial substitution of PCB with PEB in PBS and the full-sized stable chimeric PBS complexes were observed. The results of this study could contribute to efficient light harvesting and energy transfer to the photosynthetic reaction center.

## Results and Discussion

### Construction of Cyanobacterial Strains with Complete Control over PEB Levels

**Figure 2:**
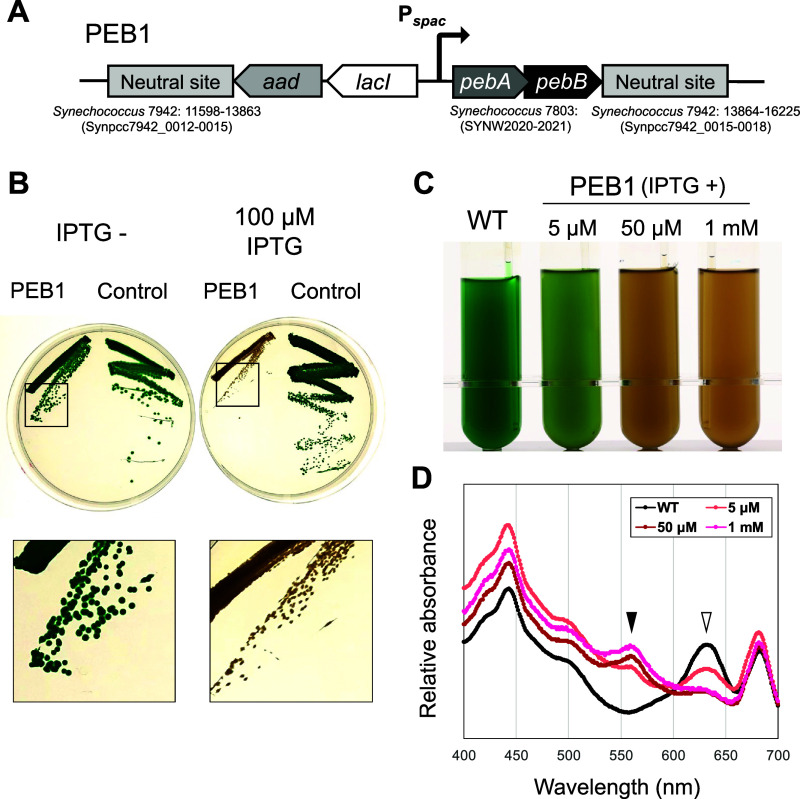
Construction of a PEB1 strain allowing control of PEB levels. (A) Schematic diagram of the genomic construct of *Synechococcus* 7942 PEB1 expressing *pebA* and *pebB* in an IPTG-dependent manner. The *pebA* and *pebB* genes of *Synechococcus* 7803 were placed under an IPTG-dependent *spac* promoter and introduced into the neutral site (NS) of *Synechococcus* 7942 along with the spectinomycin resistance gene (*aad*) and *lacI*. (B) Phenotype of the PEB1 strain on the plates. The PEB1 and control strain that transformed using the empty vector were grown for 10 days under white light (60 μmol photons m^–2^ s^–1^) on BG-11 plates containing 40 μg mL^–1^ spectinomycin with (right) and without (left) 100 μM IPTG. Enlarged images of colonies are shown below. (C) Wild-type and PEB1 strains were grown in liquid medium under white light with 5, 50, and 1000 μM IPTG. The strains were cultured in liquid BG11 in a photobioreactor at 30 °C, with air bubbling for 1 week. (D) Absorption spectra of the cultures. The spectra were normalized to 680 nm. Closed arrowhead: peak at 560 nm; open arrowhead: peak at 630 nm. PEB1, *Synechococcus* 7942 PEB1 strain; control, *Synechococcus* 7942 carrying only *aad* gene in NS; IPTG, isopropyl ß-D-1-thiogalactopyranoside; WT, wild type.

We suspected that the unstable phenotype in the previous study conducted with *Synechococcus* 7002 related to the strong and constitutive expression of PebAB.^17^ To fully regulate PEB metabolism in cyanobacteria, we selected the cyanobacterium *Synechococcus* 7942 as the experimental organism because this strain can strictly control exogenous gene expression by regulating the LacI-*lacO* system with isopropyl ß-D-1-thiogalactopyranoside (IPTG).^18,19^ In the marine cyanobacterium *Synechococcus* 7803, the ORFs of *pebA* and *pebB* partially overlap and are thought to be expressed in the same transcriptional unit. Thus, we retrieved intact ORFs from the genome of *Synechococcus* 7803, placed them under an IPTG-inducible *spac* promoter with a ribosomal binding site, and introduced them into a neutral site (NS) on the chromosome of *Synechococcus* 7942 (Figure 2A). We named the resulting strain *Synechococcus* 7942 PEB1 and examined its phenotype during *pebAB* induction. Brown colonies appeared on plates containing 100 μM IPTG under white light (WL) irradiation, whereas the green color was retained in the medium without IPTG (Figure 2B). In contrast to a previous study using *Synechococcus* 7002,^17^ no green suppressor colonies appeared in the PEB1 strain after >10 d of incubation with IPTG (Figure 2B), indicating that the PEB1 strain could stably control PEB metabolism. In the liquid medium, browning was observed in an IPTG dose-dependent manner (Figure 2C). The absorption spectra of these cultures showed a decrease in the 630 nm peak representing PC and an increase in the 560 nm peak following IPTG addition (Figure 2D), indicating that PEB bound to phycobiliproteins without using PEB lyase.

**Figure 3:**
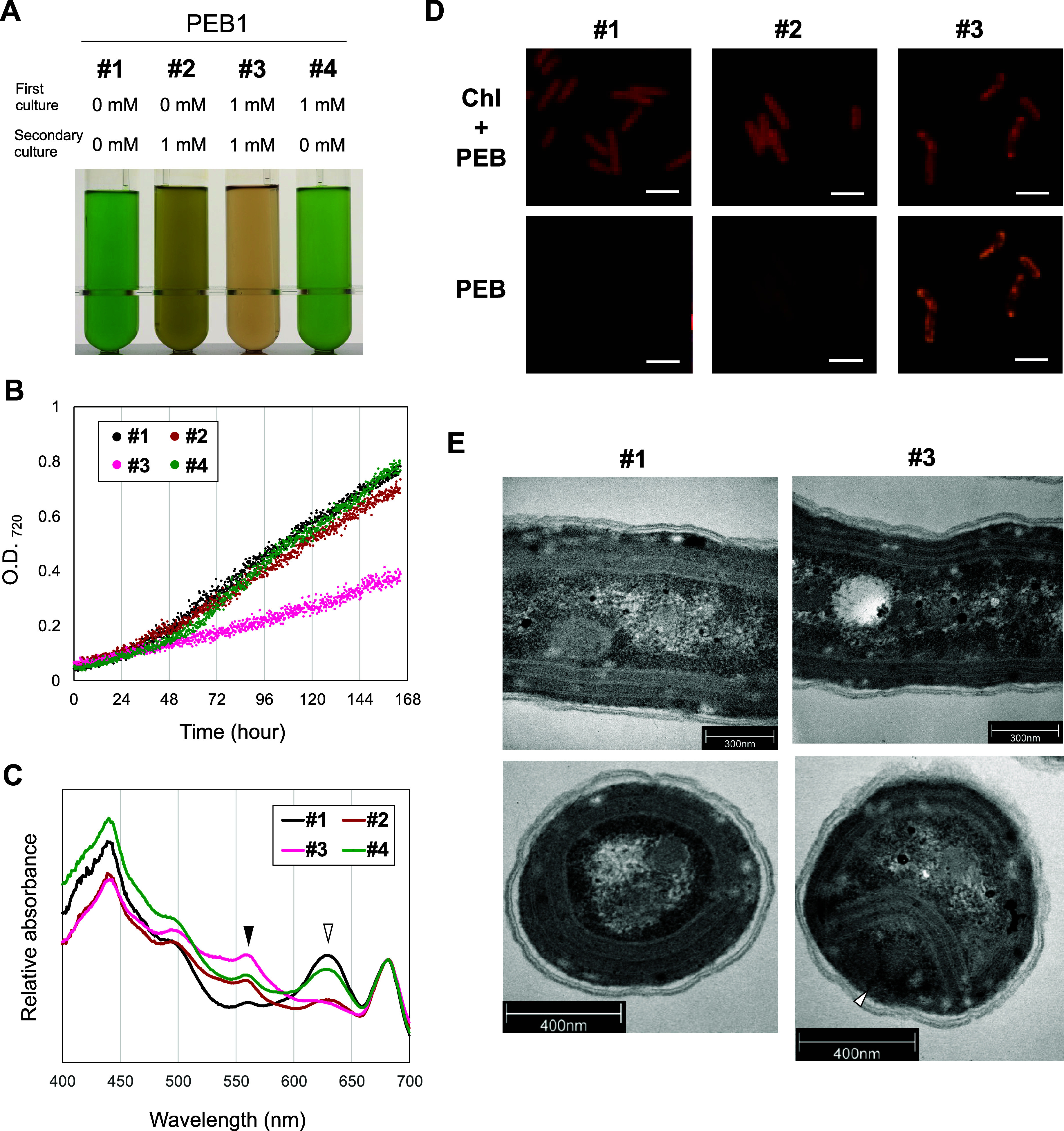
Acclimation and reversibility of the IPTG induction. (A) PEB1 cultures after 1 week of incubation in the liquid medium. (B) Growth curves of PEB1 cultures. (C) Absorption spectra of the cultures. Closed arrowhead: peak at 560 nm; open arrowhead: peak at 630 nm. The spectra were normalized to 680 nm. (D) Fluorescence microscopy of Chl and PEB fluorescence in #1–3 cells. White bar: 5 μm. (E) Transmission electron microscopy of #1 and #3 PEB1 cells. Upper panels, horizontal images; lower panels, vertical images. Open arrowhead indicates space and unusual structure observed between the cell and the thinned thylakoid membrane. PEB, phycoerythrobilin; Chl, chlorophyll; O.D., optical density.

To study acclimation and reversibility, we examined the sequential batch culture of PEB1 with or without IPTG. When green noninduced culture was incubated with 1 mM IPTG, the color of the culture was converted from green to brown after 1 week (Figure 3A). When this brown culture was inoculated in the second culture with 1 mM IPTG, the culture color was changed from brown to pink. On the other hand, the brown culture was inoculated without IPTG, and the color was fully reverted to green. (Figure 3A; #3 and #4) (Supplementary Movie). The induction of *pebAB* by the addition of IPTG caused changes in the absorption spectra and growth of PEB1 (Figure 3B, C). Compared to the green culture grown without IPTG, the brown culture after the first incubation with 1 mM IPTG for 1 week showed a decrease in the absorption peak at 630 nm corresponding to PCB-bound phycobiliproteins and an increase in the absorption peak at 560 nm corresponding to PEB-bound phycobiliproteins (Figure 3C; #2). The pink culture after the secondary incubation with IPTG had a higher absorption peak at 560 nm and a lower peak at 630 nm than the brown culture (Figure 3C; #3). Growth of the culture just after the first IPTG induction was the same as the green culture without IPTG but slightly retarded later (Figure 3B; #2). Then, the growth of the secondary culture with IPTG was severely suppressed compared with the first culture (Figure 3B; #3). These gradual changes in growth can be accounted for by the progressive replacement of PCB-bound PBS with PEB-bound PBS due to dilution with IPTG-induced PEB-bound PBS. On the other hand, the growth of the IPTG-induced culture without IPTG was slower than the noninduced culture but was later recovered (Figure 3B; #4). These results indicate that the full acclimation to 1 mM IPTG takes two sequential inductions, and the acclimation was fully canceled by one batch culture without IPTG. Immediately after 1 mM IPTG induction, the PEB1 strain contained a sufficient amount of PCB-bound PBS to grow under WL conditions. During cell proliferation with PEB production, the PCB-bound PBS was replaced with newly synthesized PEB-bound PBS, and the color of the culture changed from green to brown (Figure 3A; #2). After secondary acclimation to this process, PEB1 further decreased its absorption at 630 nm, wherein the culture turned from brown to pink (Figure 3A; #3) and reduced growth under white light (Figure 3B; #3).

To further study the growth inhibition, we investigated the effects of PEB accumulation on cell morphology (Figure 3D). Microscopic observations revealed abnormal cell morphology resulting from PEB accumulation. In the brown cells after the first IPTG culture, PEB and chlorophyll fluorescence were distributed throughout the cells, whereas the pink cells after the second IPTG culture showed that the foci of strong PEB fluorescence had an abnormal distribution of chlorophyll (Figure 3D). Transmission electron microscopy (TEM) revealed that the pink cells had an abnormal cell morphology with narrow intervals between the thylakoid membranes (Figure 3E), suggesting that the number of PBS complexes was reduced at these intervals. In addition, unusual structures were observed in the space between the cell and the thylakoid membrane (Figure 3E, open arrowhead). These unique structures could have arisen from an overproduction of PEB-bound chimeric phycobiliproteins.

### Collapse of PBS Rod Complexes because of Excessive PEB Accumulation

**Figure 4:**
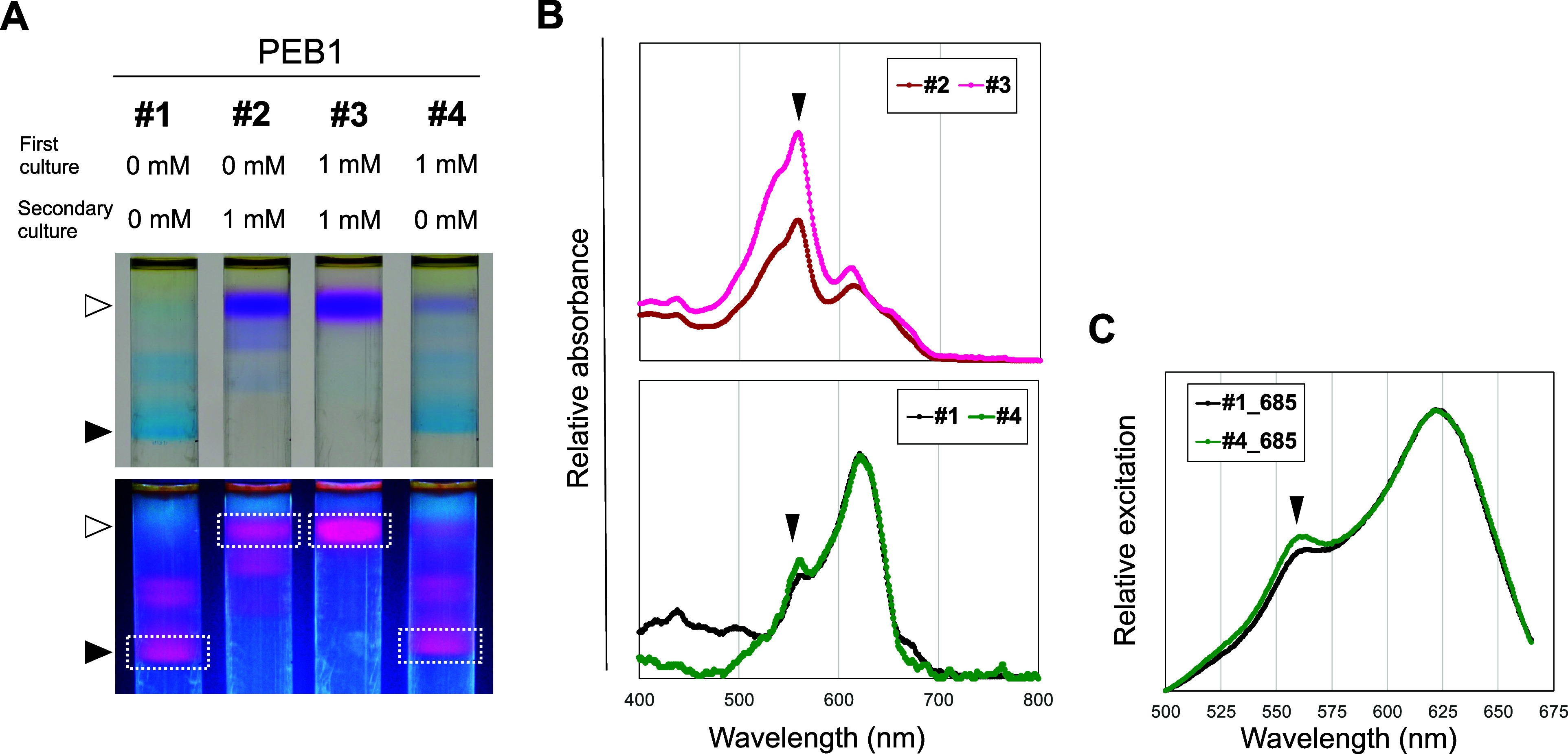
Comparison of the properties of PBS complexes fractionated by SDG centrifugation. PBS was fractionated from the cell extracts of PEB1 cultures. (A) PBS fraction images obtained after SDG centrifugation. The lower image shows the excitation of phycobiliproteins after ultraviolet-A irradiation. The fractions subjected to absorption spectroscopy (B) and excitation spectroscopy (C) are enclosed by dotted lines. (B) Absorption spectra of the phycobiliprotein fractions. The samples indicated by the open and closed arrowheads in (A) are shown in the upper and lower panels, respectively. The spectra were normalized at 630 nm. (C) Excitation spectra of the PBS fractions emitting allophycocyanin fluorescence (685 nm). Spectra of the bottom fraction containing mature PBS (closed arrowheads in A) normalized to 630 nm. Closed arrowheads indicate the peak at 560 nm.

To study the PEB-PBS biochemically, we fractionated PBS and phycobiliproteins by extraction with high salt and sucrose density gradient (SDG) centrifugation (Figure 4A) that enables us to isolate the native PBS supercomplex from wild type. The green culture before IPTG induction gave a blue band of the full-sized PBS supercomplex at the bottom layer of the gradient (Figure 4A; #1, closed arrowheads). The brown and pink cultures after the IPTG acclimation lost this PBS supercomplex and instead gave a dense blue-red band at the top of the gradient, indicative of disintegration of the PBS supercomplex (Figure 4A; #2 and #3, open arrowheads). Absorption spectra revealed that the blue supercomplex of PBS before the induction showed a small 560 nm shoulder with 630 nm peak of PCB-PC (Figure 4B; #1, bottom panel), whereas the disintegrated phycobiliproteins from the brown and pink cultures showed the prominent 560 nm peak with small 630 nm peak (Figure 4B; #2 and #3, upper panel). These results indicate that the full induction with 1 mM IPTG largely replaced PCB with PEB in the PC, resulting in complete disintegration of the PBS supercomplex.

In the green culture obtained by incubating brown culture without IPTG, the size and color of the PBS complexes returned to almost their original state: the PEB peak at 560 nm was decreased markedly and the PBS supercomplex was assembled again at the noninduced level (Figure 4B; #1 and #4). These results indicate that the excess PEB accumulation after 1 mM IPTG was biochemically reversed to the original level. The excitation spectra of the 685 nm fluorescence of the terminal emitter APC in the PBS supercomplex showed that the PBS supercomplex of green cultures had a distinct peak at 560 nm in addition to the 630 nm peak (Figure 4C). These results indicate that the PEB1 strain without IPTG accumulated a small amount of PEB that is assembled in the full PBS supercomplex and transfers short-wavelength GL energy to the PBS core.

**Figure 5:**
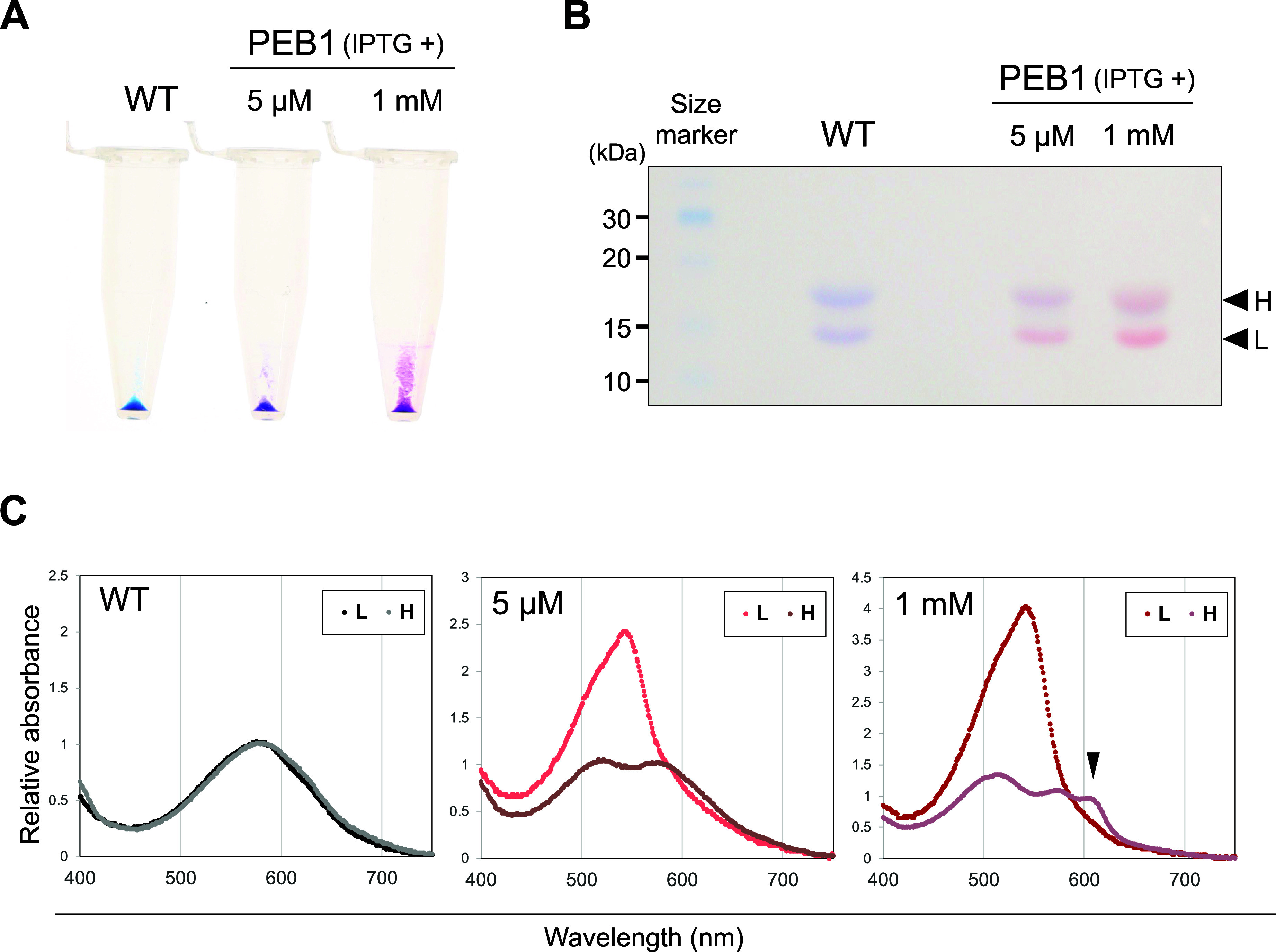
PEB preferentially binds to the C-phycocyanin alpha chain (CpcA). WT and PEB1 strains were grown under white light conditions in a liquid BG-11 medium. The indicated amounts of isopropyl ß-D-1-thiogalactopyranoside were added to the culture. (A) Pellet images of phycobiliproteins extracted from WT and PEB1 cells. (B) SDS-PAGE gel images of phycobiliproteins. Arrowheads indicate phycobiliproteins with low (L) and high (H) molecular weights containing phycocyanin and allophycocyanin subunit proteins, respectively. (C) Absorption spectra of phycobiliprotein-extracted gel. Each set of spectra was normalized to 584 nm.

Since PEB was incorporated into PBS and phycobiliproteins in PEB1, we further analyzed the binding of PEB to apoproteins. PEB has been reported to bind to CpcA^17,20,21^ and similar bindings between PEB and PBS proteins were expected in the PEB1 strain. The color of the phycobiliprotein extracts after acetone treatment suggested covalent binding of PEB to the apoproteins like the original PCB without a lyase specific to PEB (Figure 5A). When separated by sodium dodecyl sulfate (SDS)-polyacrylamide gel electrophoresis (PAGE), extracts of the PEB1 strain gave two red color bands that correspond to the blue bands of PCB-PC of wild type (Figure 5B). Proteins were extracted from each band, and the absorption spectra were compared. The absorption wavelength of the lower band shifted to a shorter wavelength in the PEB1 strain (Figure 5C), suggesting a preferential binding of PEB to CpcA *in vivo*. This is consistent with previous findings that PEB can bind to CpcA in *Synechococcus* 7002 and *Synechocystis* sp. PCC 6803 (*Synechocystis* 6803).^17,20,21^ The higher molecular weight fraction containing CpcB revealed two peaks that correspond to the PEB-PC and PCB-PC under both IPTG conditions (Figure 5C). Because CpcB has two bilin-ligating Cys residues, the two peaks may suggest that only one Cys residue could ligate PEB and the other residue does not ligate PEB. This was another peak at a longer wavelength than PCB in the “CpcB” band (Figure 5C, arrowhead) which may contain PBS core components such as allophycocyanin subunits. On the other hand, binding of DHBV, which is the PEB biosynthesis intermediate, is very unlikely because all known PBS components do not bind DHBV which has a vinyl group at C3 in contrast to the ethylidene group of PCB and PEB (Figures 1 and S2). Anyway, it should be noted that CpcB could bind PEB in *Synechococcus* 7942. This is quite a contrast to the absence of PEB binding to CpcB in *Synechococcus* 7002.^17^

### Establishment of GL-Utilizing PBSs by Controlling PEB Levels

**Figure 6:**
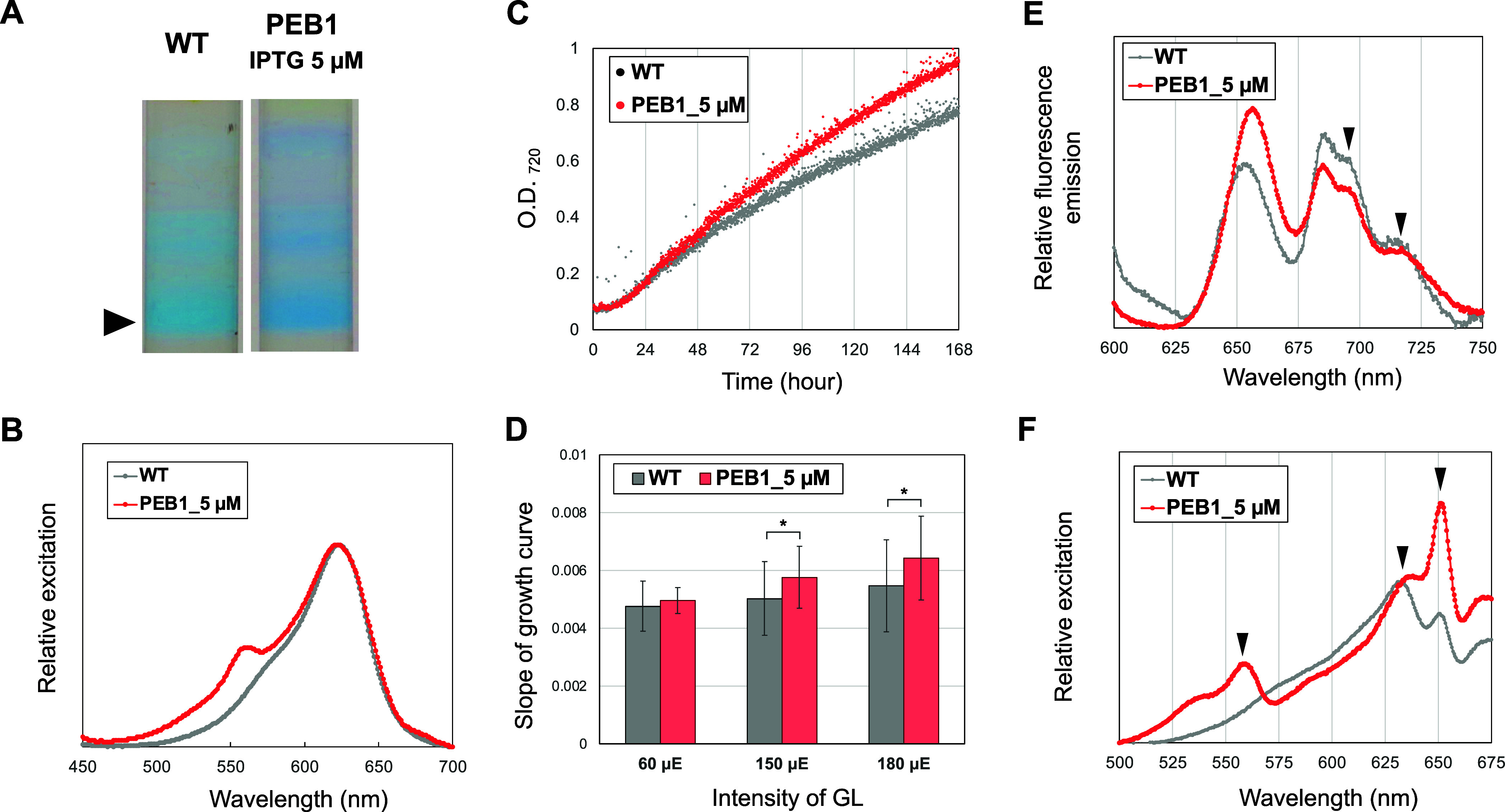
GL acclimation of PEB1. (A) PBS fraction images after sucrose density gradient centrifugation. The samples were prepared from cells grown under white light. IPTG (5 μM) was applied to PEB1 cultures to express the appropriate amounts of 15,16-dihydrobiliverdin:ferredoxin oxidoreductase (PebA) and PEB:ferredoxin oxidoreductase (PebB). (B) Excitation spectra of the bottom fractions are indicated by the closed arrowhead in (A). (C,D) Comparison of growth under GL irradiation (180 μmol photons m^–2^ s^–1^). (C) Growth curves of WT and PEB1 cultures. (D) Comparison of estimated growth rate based on the slopes of the growth curves. The intensities of the irradiated GL are shown on the *x*-axis. For statistical evaluation, *p*-values were calculated using the paired *t*-test in Microsoft Excel, *p < 0.05. (E,F) 77K fluorescence and excitation spectra of WT and PEB1 cultures. (E) Fluorescence spectra were excited at a wavelength of 530 nm. The spectra were normalized to 720 nm. The peaks of fluorescence emitted by photosystem (PS) II (695 nm) and PSI (715 nm) are indicated by arrowheads. (F) Excitation spectra measuring fluorescence at 695 nm. The spectra were normalized to 630 nm. The peaks of the absorption of 560, 630, and 650 nm are indicated by arrowheads.

We next investigated the properties of the PBS complexes and the utilization of GL in PEB1 because the PEB1 strain was expected to form full-sized PEB-bound PBS complexes at low levels of *pebAB* induction. Full-sized PBS complexes were isolated from the PEB1 strain with low *pebAB* induction by adding 5 μM IPTG. The fluorescence excitation spectrum of the isolated PBS complex revealed an additional peak at 560 nm that was not observed with the WT strain (Figure 6A, B). The growth between PEB1 (supplemented with 5 μM IPTG) and the WT strains was compared using a photobioreactor under GL. The results showed that PEB1 with 5 μM IPTG grew faster than the WT strain (Figure 6C). Comparing the slope of the growth curve (i.e., growth rate) at several light intensities with that of the WT, PEB1 showed a growth advantage under GL at light intensities greater than 150 μmol photons m^–2^ s^–1^ (Figure 6D). The energy transfer of the PEB1 culture using 77 K fluorescence spectra indicated that PEB1 was more efficient than the WT at utilizing GL. The fluorescence emitted by photosystem (PS)II (695 nm) and PSI (715 nm) was observed following excitation with GL (Figure 6E). Furthermore, the 77 K excitation spectrum of PSII (695 nm) showed peaks corresponding to the absorption of PEB-PC, PCB-PC, and PCB-APC at 560, 630, and 650 nm, respectively, indicating the ability of PEB-bound PBS to transfer GL energy to PSII (Figure 6F).

### Growth Advantage of PEB1 Strain Under GL

**Figure 7:**
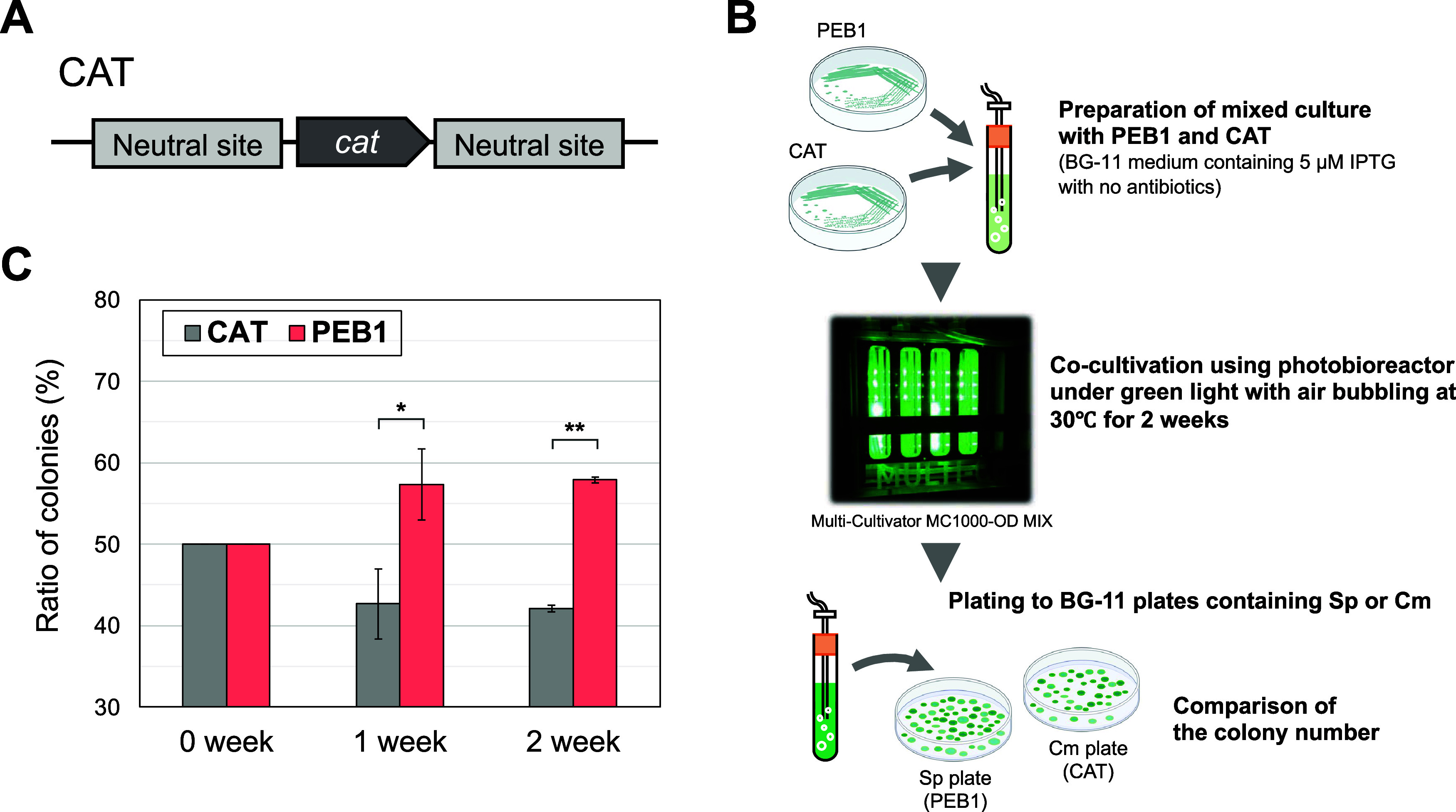
Competition assay. (A) Schematic diagram of the genomic construct of CAT strain. (B) Scheme of a competitive assay of two cyanobacteria, the PEB1 strain and the CAT strain. The scheme was drawn using elements from the BioRender platform (https://biorender.com/). (C) Populations of CAT and PEB1 strains. PEB1 was grown with CAT under GL irradiation (180 μmol photons m^–2^ s^–1^). Before and after the incubation, the culture was spread on BG-11 plates containing spectinomycin or chloramphenicol and incubated for 1 week. The populations of CAT and PEB1 strains were estimated based on the number of colonies appearing on the plates. For statistical evaluation, *p*-values were calculated using the paired *t*-test in Microsoft Excel, *p < 0.05; **p < 0.01. CAT, *Synechococcus* 7942 CAT strain.

We performed a competition assay to examine the advantages of PEB-bound PBS in the PEB1 strain under GL conditions. The chloramphenicol acetyltransferase gene (*cat*) was inserted into an NS on the chromosome of the WT and the resultant strain was named *Synechococcus* 7942 CAT (Figure 7A), which was expected to have the same GL utilization capacity as the WT strain. The CAT strain was co-inoculated with PEB1 in a BG-11 liquid medium containing 5 μM IPTG without antibiotics and cultured under 180 μmol photons m^–2^ s^–1^ GL (Figure 7B). Before and after 1- and 2-week competition under GL, the culture was spread onto BG-11 medium plates containing spectinomycin or chloramphenicol. After screening, the number of colonies on the plates was counted, and the population of each strain was calculated (Figure 7B). The results showed a significant increase in the population of the PEB1 strain compared with that of the CAT strain at both 1 and 2 weeks (Figure 7C), indicating that the PEB1 strain showed better growth than the CAT strain under GL.

### Functional Model of PEB-Bound PBS in PEB1

**Figure 8:**
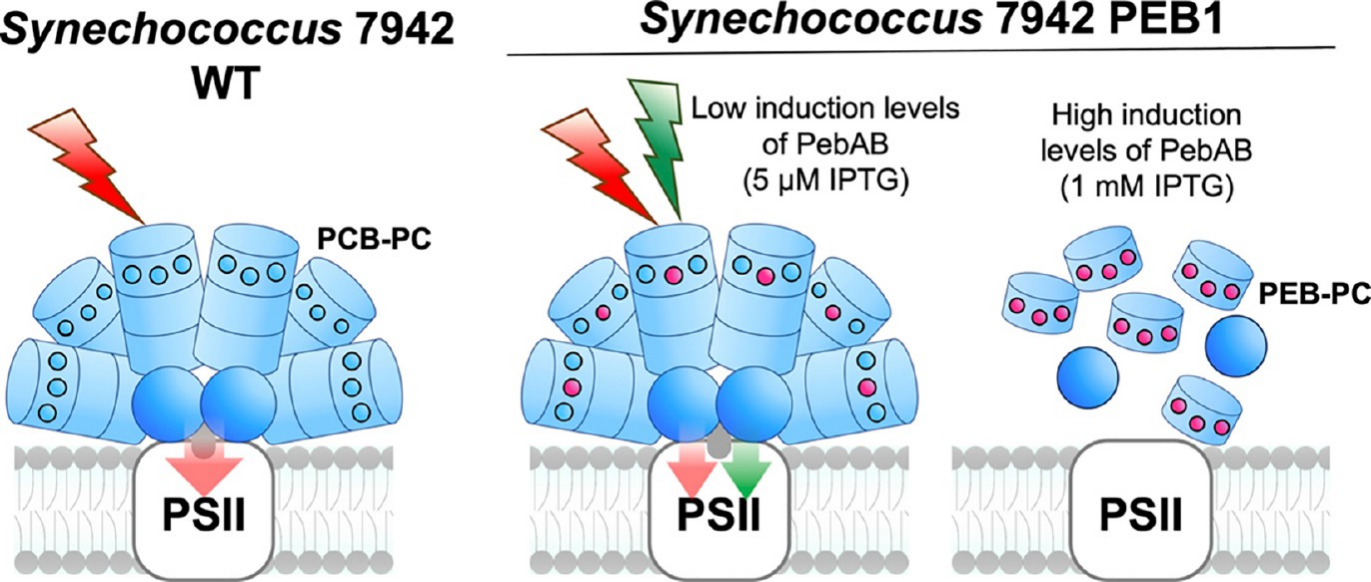
Hypothetical model of PBS during the induction of PEB. Hypothetical model of PBS in *Synechococcus* 7942 WT and PEB1 at high and low PEB induction levels. Bilins (PCB and PEB) that bound to the outer disk are shown as blue (PCB) or red (PEB) circles. PCB-PC, disk complex composed of CpcA, CpcB, and PCB; PEB-PC, disk complex composed of CpcA, CpcB, and PEB; PSII, photosystem II.

The proposed model of the effects of PEB production is shown in Figure 8. The *Synechococcus* 7942 WT strain, which used PCBs in the PBS, efficiently transferred red light to the PS. In the PEB1 strain, PBS disassembly occurred at high PEB induction levels, whereas at low levels, PBS was constructed to absorb both red light and GL and transfer the light energy to the PSs. It should also be mentioned that *Synechococcus* 7942 is a versatile platform to engineer PBS for synthetic biology with respect to the following at least two points: the technology that can precisely control the expression of exogenous genes is well established,^18,19^ and it assembles functional PBS even in bilin lyase mutants compared with *Synechococcus* 7002.^22,23^

In nature, several cyanobacteria have been found to flexibly and dynamically rearrange their photosynthetic apparatus, including PBS, in response to the light environment.^24^ These changes are accomplished through a complex cellular response involving many genes that adapt PBS to the light environment, including signaling systems, bilin synthases, apoproteins, and lyases. In this study, we artificially altered the properties of PBS by manipulating PEB metabolism. To our knowledge, this is the first study to construct a cyanobacterium that can utilize GL to accelerate growth by genetic modification. The accumulation of small amounts of PEB-based PCs under precise control is the key to this achievement. The expression of additional factors such as PEB-binding apoproteins and other components of PEB-PBS may allow the creation of cyanobacteria with PBSs, which would be more suitable for differing light conditions.

Our study provides an experimental basis for future research to elucidate the underlying mechanisms governing PBS adaptation and offers potential applications in bioengineering, synthetic biology, and renewable energy research. Recent attempts to modify the bilin metabolism of *Synechocystis* 6803 have highlighted the need to understand the unique regulation of PBS in diverse cyanobacteria.^20^ This study deepens our understanding and aids in the development of artificial pigments. In addition, this research is at the forefront of advancing the development of these pigments. Our study of PEB-based PCs, which are powerful fluorescent emitters, has the potential to revolutionize industrial applications using novel fluorescent reagents. In particular, greater accumulation of the pink-colored component, PEB-PC, at high IPTG concentrations (Figure 4A; 2# and 3#) is advantageous for the production of fluorescent reagents. Compared with natural PE synthetic cyanobacteria, model cyanobacteria such as *Synechococcus* 7942 offer faster growth rates and easier manipulation. Hence, this pioneering synthetic pathway holds great promise for enhancing phycobiliprotein production.

## Conclusions

In conclusion, we successfully achieved precise and stable control of PEB levels in *Synechococcus* 7942 by regulating the expression of *pebAB.* In addition, we demonstrated the effect of PEB in PCB-bound PBS. Our results showed that the overproduction of PEB led to PBS disintegration in *Synechococcus* 7942, underscoring the necessity of an optimal bilin-apoprotein combination to maintain PBS structural integrity. In contrast, low PEB levels caused the formation of a stable chimeric PBS complex with partial substitution of PCB by PEB. Cells harboring this PCB-PEB chimeric PBS exhibited enhanced utilization of GL for photosynthetic electron transfer and displayed accelerated proliferation rates compared with the WT strain under GL conditions. These findings improve our understanding of PBS and highlight the structural importance of the bilin composition while providing a basis for future studies on PBS adaptation in the fields of bioengineering, synthetic biology, and renewable energy.

## Methods

### Culture Conditions for Cyanobacteria

The cyanobacterium *Synechococcus* 7942 WT strain and its derivatives were grown photoautotrophically at 30 °C under continuous WL illumination (60 μmol photon m^–2^ s^–1^) in a modified BG-11 medium containing double the usual amount of sodium nitrate (final concentration = 35.3 mM) and 20 mM 4-(2-hydroxyethyl)-1-piperazineethanesulfonic acid (HEPES)-KOH (pH 8.2) with 2% CO_2_ bubbling. When appropriate, spectinomycin (final concentration = 40 μg mL^–1^) and chloramphenicol (final concentration = 10 μg mL^–1^) were added to the media.

The growth experiments were performed using a Multi-Cultivator MC1000-OD STANDARD and MIX (Photon Systems Instruments, Czech Republic) under continuous illumination with cool WL (425–725 nm; 60 μmol photons m^–2^ s^–1^) or GL (530 nm; 60, 150, and 180 μmol photons m^–2^ s^–1^) and bubbling ambient air. The emission spectra of the WL and GL are shown in Figure S1. After preincubation on a BG-11 medium plate for 1 week, the cells were harvested, inoculated into liquid BG-11 medium with IPTG at an optical density (OD)_720_ of 0.05, and cultivated for 1 week. An approximate growth curve was constructed based on the OD value up to day 7 (168 h). Measurements were conducted in triplicate and the slope of the approximate growth curve was compared with the growth rate. Significance was determined using a paired *t*-test in Microsoft Excel.

### Strain Construction

A polymerase chain reaction (PCR) was used to amplify the DNA fragments of the PEB synthase genes *pebA* and *pebB* from *Synechococcus* 7803 (open reading frame ID: SYNW2020 and 2021), upstream and downstream of the NS of *Synechococcus* 7942 genomic region, and spectinomycin-resistant gene (*aad*) with *lacI–spac* promoter unit using PrimeSTAR DNA polymerase (TaKaRa, Shiga, Japan) and the appropriate primer sets (F1/R2, F3/R4, F5/R6, F7/R8, and F9/R10; Table S1). Five DNA fragments were recombined by PCR using the primer set F3/R6 (Table S1), and the resulting fragment was introduced into *Synechococcus* 7942 to obtain spectinomycin-resistant transformants. Successful transformation of the strains with DNA fragments was confirmed by PCR amplification using the specific primer set F11/R12, and the resulting strain was named *Synechococcus* 7942 PEB1.

The competitor strain *Synechococcus* 7942 CAT, containing a chloramphenicol-resistant gene (*cat*) at the NS, was constructed and used for the growth competition test under GL. Three DNA fragments containing *cat*, upstream and downstream of NS, were amplified using the appropriate primer sets (F13/R14, F3/R15, and F16/R6; Table S1) and recombined by PCR using the primer set F3/R6. The resulting fragment was then introduced into *Synechococcus* 7942 to obtain chloramphenicol-resistant transformants. Successful transformation of the strains was confirmed by PCR using the primer set F11/R12.

### Fluorescence Microscopy

Fluorescent images were obtained using a BX53 microscope (OLYMPUS, Tokyo, Japan) at 100× magnification with a DP71 digital camera (OLYMPUS) and DP Controller software ver. 3.3.1.292 (OLYMPUS). Chlorophyll/PEB and PEB autofluorescence were observed using U-MWIG3 and U-FRFP filter units (OLYMPUS), respectively.

### TEM

The PEB1 strain was inoculated in the BG11 liquid medium at an OD_750_ of 0.1, with and without 1 mM IPTG, and incubated for 24 h at 30 °C. The cells were collected by centrifugation at 3000 × *g* for 10 min at 25 °C and fixed with 1 mL of ice-cold 2% glutaraldehyde solution overnight at 4 °C (prefixation). The cells were collected by centrifugation and washed with 1 mL of 0.1 M phosphate buffer (pH 7.4) overnight at 4 °C. For postfixation, ice-cold 2% osmium tetroxide was added to the samples, which were then incubated for 3 h at 4 °C. The stained cells were dehydrated with increasing concentrations of ethanol (50, 70, 90, and 100%) for 15 min each, and the dehydrated cells were embedded in a gelatin capsule with epoxy resin for 2 days at 60 °C. Ultrathin sections (80–90 nm) stained with 2% uranyl acetate for 15 min and lead staining solution for 2 min, were subjected to TEM (JEM1200EX; JEOL, Tokyo, Japan) at the Hanaichi Ultra Structure Research Institute (Aichi, Japan).

### Analysis of Phycobiliproteins

The cells were harvested and resuspended in 100 μL of A buffer (containing 10% glycerol, 100 mM NaCl, and 20 mM HEPES-NaOH; pH 7.5) and disrupted with glass beads using a bead beater (Micro Smash MS-100R, TOMY Seiko Co., Tokyo, Japan). After centrifugation at 20,000 × *g* for 1 min at 18 °C, 60 μL of supernatant was added to fresh tubes and mixed with 240 μL of acetone (final concentration = 80%) to remove chlorophyll and carotenoids from the cell lysates. The resulting samples were centrifuged at 20,000*g* for 1 min and pellets containing phycobiliproteins were obtained. The binding of bilins to apoproteins was analyzed using SDS-PAGE. Subsequently, 20 μL of supernatant mixed with loading dye (final concentration = 0.0625 Tris-HCl, 10% glycerol, 2% SDS, and 0.01% bromophenol blue) was separated on a 15% (w/v) polyacrylamide gel. After PAGE, the excised gels were extracted using ATTOPREP MF (ATTO, Tokyo, Japan), and the absorption spectra were obtained.

### Isolation of PBSs by SDG Centrifugation

The *Synechococcus* 7942 culture (OD_750_ = 1.0; 30 mL) was harvested by centrifugation (3000 × *g*, 10 min, 25 °C). After washing with 1 mL of 0.6 M potassium phosphate (KP) buffer (pH = 7.0), the cells were again centrifuged (3000 × *g*, 10 min, 25 °C) and maintained in a freezer for further analysis. The cells were washed twice in 0.6 M KP buffer and resuspended in 0.6 mL of 0.6 M KP buffer, after which they were lysed by vortexing with glass beads, and the PBS complexes were extracted from the thylakoid membranes by vortexing with Triton X-100 (final concentration = 2%) for 15 min. After centrifugation (20,000 × *g*, 20 min, 18 °C), 200 μL of supernatant was loaded onto sucrose gradients (10–50% sucrose in 0.6 M KP buffer) prepared in tubes (14 × 89 mm, Open-Top Thinwall Ultraclear tubes [Beckman Coulter, CA, USA]) using a Gradient Master (Beckman Coulter). The gradients were centrifuged at 154,300 × *g* for 18 h at 18 °C (SW41Ti rotor, Optima XE-90 Ultracentrifuge [Beckman Coulter]). After centrifugation, the samples were irradiated with ultraviolet (UV)-A (365 nm) using a UV lamp (UVP Inc.) to observe the PC and PE fluorescence of the phycobiliproteins.

### Spectrometry

The absorption spectra of the cultures, phycobiliproteins, and PBS complexes were measured at 25 °C using a spectrophotometer (model UV-1800, Shimadzu, Japan). The excitation spectra were recorded at 685 nm (APC) using a spectrophotometer (FP-8200; JASCO, Japan). To measure the 77 K fluorescence excitation spectra, the samples were frozen in liquid nitrogen and PSII fluorescence (695 nm) was measured (model RF-6000, Shimadzu, Japan).

### Growth Competition Test under GL

After preincubation on BG-11 medium plates containing spectinomycin (for PEB1) or chloramphenicol (for CAT), the PEB1 and CAT cultures were inoculated in the BG11 liquid medium containing 5 μM IPTG without antibiotics at an OD_750_ of 0.025 and incubated for 2 weeks at 30 °C under continuous GL (530 nm, 180 μmol photons m^–2^ s^–1^) with bubbling ambient air. Immediately after inoculation and 1 and 2 weeks subsequent, 10 μL of the culture medium was incubated on plates containing spectinomycin or chloramphenicol, and the number of colonies that grew on each plate after 1 week of incubation was counted. Measurements were conducted in triplicate, and the data are presented as mean ± standard deviation (SD). Significance was determined using a paired *t*-test in Microsoft Excel.

## Supporting Information Available

Supplemental figure and lists of primers (PDF)
Color change of PEB1 cultures over time (MP4)

## Abbreviations

PBS: phycobilisome
PC: phycocyanin
PE: phycoerythrin
PEC: phycoerythrocyanin
APC: allophycocyanin
PCB: phycocyanobilin
PEB: phycoerythrobilin
PSII: photosystem II
PSI: photosystem I
NS: neutral site
IPTG: isopropyl ß-D-1-thiogalactopyranoside
SDG: sucrose density gradient
WL: white light
GL: green light
