# Supplementary material for: Functional Modification of Cyanobacterial Phycobiliprotein and Phycobilisomes through Bilin Metabolism Control
Source: ACS Synth Biol. 2024 Jul 22;13(8):2391–401. doi: 10.1021/acssynbio.4c00094 (PMC11334911; doi:10.1021/acssynbio.4c00094)
Supplement: Supplementary file 1 — sb4c00094_si_001.pdf [file sb4c00094_si_001.pdf]

Supporting Information

Functional Modification of Cyanobacterial Phycobiliprotein and Phycobilisomes by Pigment Metabolism control

Mizuho Sato, Takeshi Kawaguchi, Kaisei Maeda, Mai Watanabe, Masahiko Ikeuchi, Rei Narikawa, and Satoru Watanabe*

*** Correspondence:** Corresponding Author: s3watana@nodai.ac.jp

1 Movie

Supplementary Movie. Color change of PEB1 cultures over time (mp4. file)

To prepare the primary cultures, *Synechococcus* 7942 PEB1 strain harvested from BG11 plates were inoculated into BG11 liquid medium with and without 1 mM IPTG and incubated for 3 days. Those cultures were further transferred to medium with and without 1 mM IPTG at OD=0.2 as secondary cultures and monitored for color change over 36 hours. The movie, compressed to one second per hour, shows 36 hours of recording.

2 Table

Table S1. Oligonucleotide primers used in this study.

Primer name	Sequence (5′ to 3′)[a]
Strain construction	
F1	CTTCAATCACTAGAGCTACGACAAGGAAGTTGAGGCGGC
R2	GAGGAGAAATTAACTATGTTCGACTCTTTCCTCAAC
F3	CGCGAATCCAGAACTTTGATGTCC
R4	ACTTCCTTGTCGTAGCTCTAGTGATTGAAGGGGCCTGC
F5	CTTCTCTCAATTAGCTCCACCGATGTAGCGGTC
R6	CGATCGTCAAAGGTGAGTAGCCG
F7	GAAAGAGTCGAACATAGTTAATTTCTCCTCTTTAATGAATTCAA
R8	CTGCGCTTTTTTTCATCACTGCCCGCTTTCCAGTCGG
F9	GAAAGCGGGCAGTGATGAAAAAAAGCGCAGCTGAAATAG
R10	GCTACATCGGTGGAGCTAATTGAGAGAAGTTTCTATAGAATTTTTC
F11	GAAGTGGCGATCGCCGTGATCG
R12	GATCAACACGGTGCAGGGTGG
F13	CTTCAATCACTAGAGCTGCGTTAGTCGTCATTAAGCA
R14	GCTACATCGGTGGAGTTATAAAAGCCAGTCATTAGGCCTATCTGAC
R15	TGACGACTAACGCAGCTCTAGTGATTGAAGGGGCCTGC
F16	GACTGGCTTTTATAACTCCACCGATGTAGCGGTC

3 Supplementary Figure

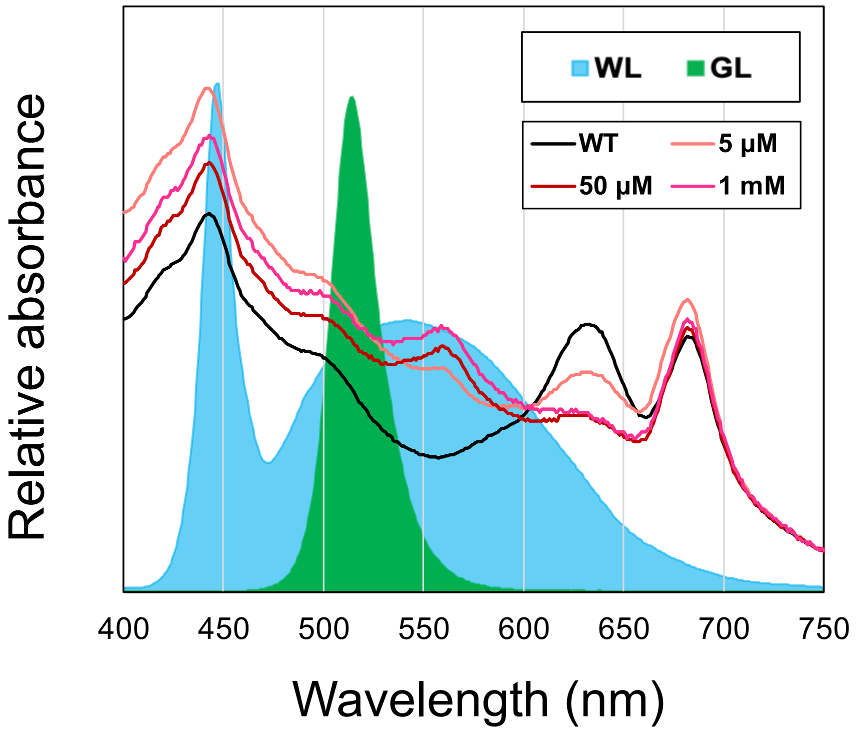

Figure S1. Spectrum of LEDs used for growth test.

LED spectra (WL, white light; GL, green light) of Multi Cultivator MC1000-OD are shown along with the absorption spectra of *Synechococcus* 7942 PEB1 described in Figure 2D.

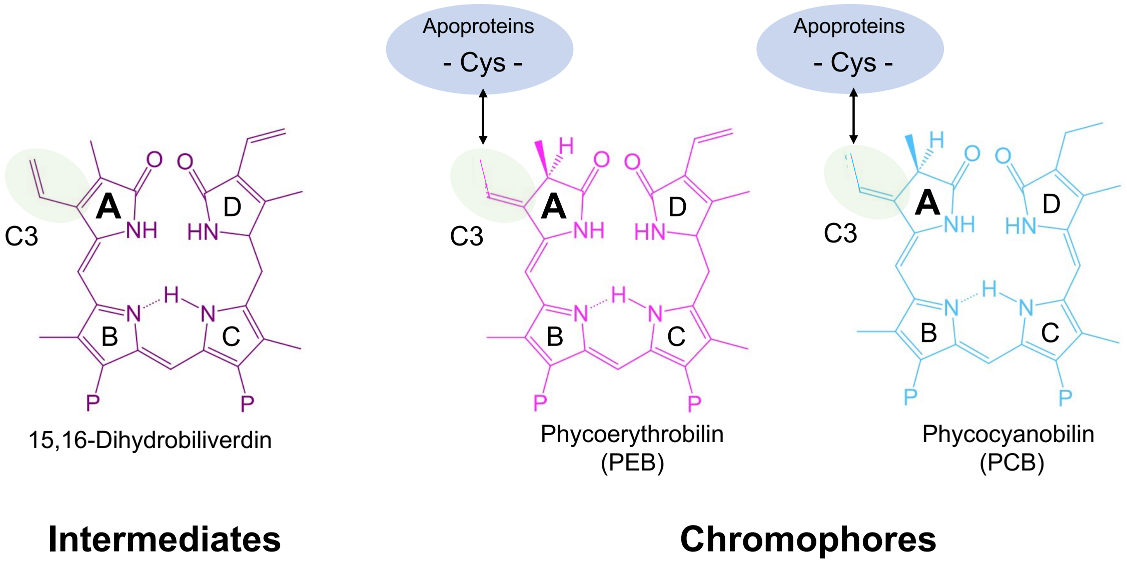

Figure S2. Structural comparison of bilins and intermediates.

C3 portion of the A ring is necessary for the binding between bilins and apoproteins. The intermediates 15,16-DHBV do not work as chromophore, since it cannot interact with apoproteins.
